# Optogenetic control of nerve growth

**DOI:** 10.1038/srep09669

**Published:** 2015-05-18

**Authors:** Seongjun Park, Ryan A. Koppes, Ulrich P. Froriep, Xiaoting Jia, Anil Kumar H. Achyuta, Bryan L. McLaughlin, Polina Anikeeva

**Affiliations:** 1 Department of Mechanical Engineering, Massachusetts Institute of Technology, Cambridge, Massachusetts 02139, United States; 2 Research Laboratory of Electronics, Massachusetts Institute of Technology, Cambridge, Massachusetts 02139, United States; 3 Department of Materials Science and Engineering, Massachusetts Institute of Technology, Cambridge, Massachusetts 02139, United States; 4 Charles Stark Draper Laboratory, 555 Technology Square, Cambridge, MA 02139, United States; 5 Simons Center for the Social Brain, Massachusetts Institute of Technology, Cambridge, Massachusetts 02139, United States

## Abstract

Due to the limited regenerative ability of neural tissue, a diverse set of biochemical and biophysical cues for increasing nerve growth has been investigated, including neurotrophic factors, topography, and electrical stimulation. In this report, we explore optogenetic control of neurite growth as a cell-specific alternative to electrical stimulation. By investigating a broad range of optical stimulation parameters on dorsal root ganglia (DRGs) expressing channelrhodopsin 2 (ChR2), we identified conditions that enhance neurite outgrowth by three-fold as compared to unstimulated or wild-type (WT) controls. Furthermore, optogenetic stimulation of ChR2 expressing DRGs induces directional outgrowth in WT DRGs co-cultured within a 10 mm vicinity of the optically sensitive ganglia. This observed enhancement and polarization of neurite growth was accompanied by an increased expression of neural growth and brain derived neurotrophic factors (NGF, BDNF). This work highlights the potential for implementing optogenetics to drive nerve growth in specific cell populations.

Following traumatic injury, functional recovery of the peripheral nervous system (PNS) is impeded by cellular debris, scarring, and tardy axonal growth^1^,^2^, and injury gaps exceeding 4 cm often require surgical intervention^3^,^4^. The ‘gold-standard’ for nerve repair, autografts, as well as FDA-approved synthetic nerve guidance channels yield limited success for larger injury gaps, leaving patients with long-term disabilities^5^,^6^. Thus, a clinical need exists for new strategies to promote axonal regeneration and re-myelination.

To overcome the regenerative barriers such as inhibitory myelin and scarring, and to increase the rate of axonal growth, numerous strategies have been investigated. Presentation of neurotrophic factors^7^,^8^, geometric constraints^9^,^10^, supportive cell grafts (Schwann cells^11^ or stem cells^12^), chemical gradients^13^,^14^,^15^, and topographical cues^16^ have been shown to influence neurite outgrowth *in vivo* and *in vitro*. In addition, stimulation with direct and alternating current (DC and AC) electrical fields enhances neurite sprouting and growth^17^. Al-Majed and colleagues observed that one hour of AC electrical stimulation increased neural regeneration *in vivo*^18^, which was correlated with a heightened expression of brain-derived neurotrophic factor (BDNF), TrkB receptor, and GAP-43^19^. Similarly *in vitro*, a DC electric field applied for 8 hours was shown to increase neurite outgrowth, Schwann cell proliferation and migration, and expression of nerve growth factor (NGF)^20^. While promising as a means for enhancing regeneration following PNS injury, electrical stimulation in the context of nerve growth remains poorly understood due to its lack of cell-type specificity and incompatibility with concomitant electrophysiological recordings.

Optogenetics allows for temporally precise excitatory and inhibitory control of neural activity in genetically distinct cell populations expressing light-gated ion channels, such as channelrhodopsin 2 (ChR2) and halorhodopsin (NpHR)^21^,^22^. While primarily exploited for manipulating neuronal activity in the brain^23^,^24^, optogenetics has been applied in PNS for optical recruitment and blocking of lower limb muscle activity^25^ as well as in a spinal cord injury rodent model to rescue respiratory function^26^. Most recently, opsins have been introduced into pluripotent stem cells, which following engraftment into a transected sciatic nerve has enabled the restoration of neural circuitry and manifested in optical control of muscle contractions^27^.

Motivated by the regenerative promise of electrical stimulation, we explored optogenetics as a means to promote neurite growth. Using light sensitive whole dorsal root ganglia (DRGs) from transgenic *Thy1-ChR2-YFP* mice expressing ChR2^28^, we tested the hypothesis that optically induced neural activity will increase neurite outgrowth. We assessed a wide range of optical stimulation frequencies and durations, and ultimately correlated the enhancement of outgrowth to the total number of stimulation pulses. Furthermore, in co-cultures of wild-type (WT) and ChR2-expressing DRGs, we found increased and directionally biased outgrowth of optically sensitive neurites, exemplifying the cell-specific targeting of optogenetics. Directional bias was also observed in the outgrowth of WT DRGs in the presence of stimulated ChR2-DRGs, which may be attributed to the increased secretion of NGF and BDNF from the latter. Taken together, our findings suggest that optogenetics may serve as a tool to study the underlying mechanisms of neural regeneration, thus informing future approaches to improve functional recovery following PNS injury.

## Results

### Neurite outgrowth increased with optical stimulation

**Figure 1:**
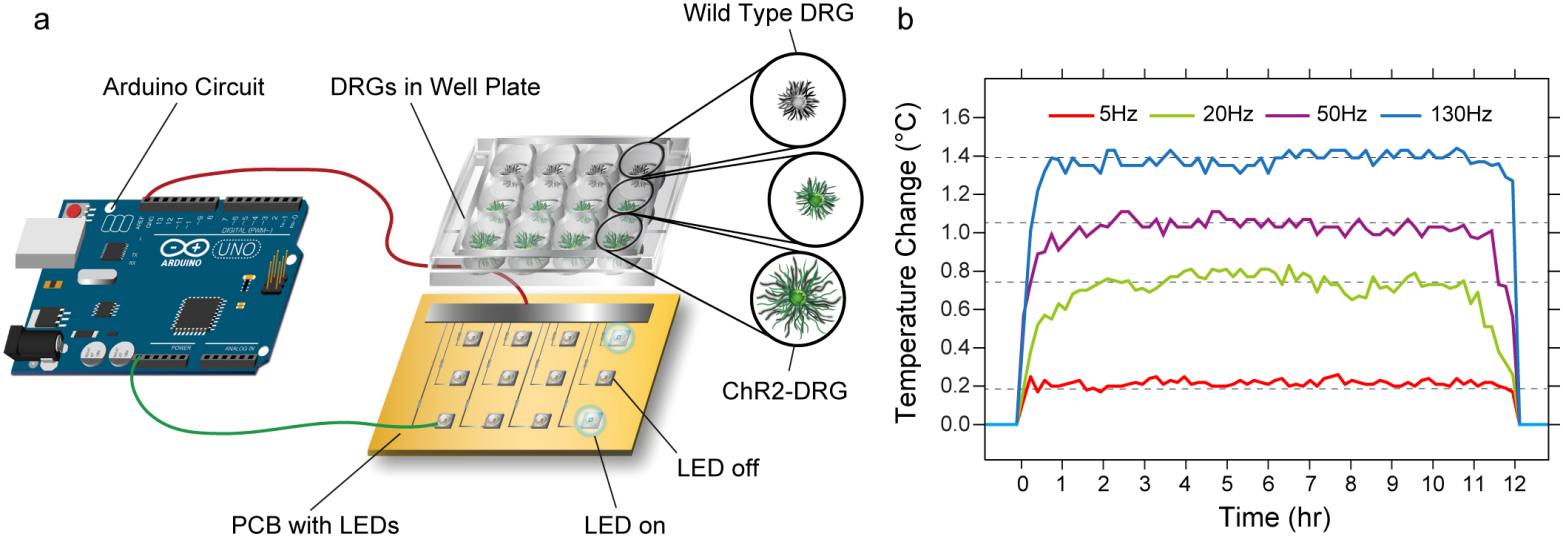
The design of a custom LED array for optical stimulation. (a) A schematic demonstrating the LED array design and the experimental setup for optical stimulation. The blue light LED (465 nm) array was powered and driven by an Arduino circuit. Optically stimulated ChR2 DRGs were compared to unstimulated ChR2-DRGs as well as stimulated WT DRGs. (b) Media temperature did not substantially increase above 37°C during optical stimulation at 5 Hz, 20 Hz, 50 Hz, and 130 Hz. Solid lines represent raw temperature traces and dashed lines denote average temperature values for each case.

To deliver a broad range of optical stimuli, a computer-controlled custom assembled array of light-emitting diodes (LEDs) (Fig. 1a) was engineered to interface with standard tissue culture plates and to operate in physiological conditions (37°C, 5% CO_2_, 95% Relative Humidity). LEDs were chosen to deliver 465 nm light pulses (close to ChR2 excitation peak wavelength λ = 473 nm) at optical powers of 5.5–6 mW/mm^2^ to incubated DRG cultures consistent with the threshold for ChR2-facilitated neural excitation *in vitro* (>1 mW/mm^2^)^29^. Illumination with pulsed blue light induced a negligible increase (0.2–1.4°C) in overall media temperature (Fig. 1b), preventing any thermally induced effects on the neurite outgrowth^30^.

We first applied our LED array to investigate the role of optical stimulation on neural growth. To ensure robust expression of ChR2 in our experiments, we utilized DRGs from *Thy1-ChR2-YFP* transgenic mice broadly expressing the opsin across central and peripheral nervous systems. Motivated by previous work exploring AC electrical stimulation, we applied optical stimulation at a frequency of 20 Hz for a duration of 1 hr. Stimulation was delivered in 1 second bursts separated by 1 second rest epochs to avoid potential network desensitization or neurotransmitter depletion^31^,^32^. Consistent with previous reports, a pulse width of 5 ms was used to achieve robust optical excitation of neural activity in ChR2-expressing neurons^29^.

**Figure 2:**
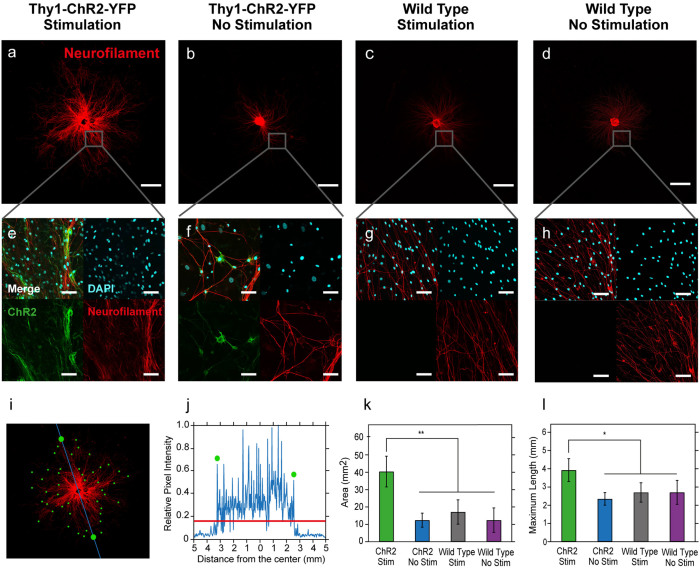
Neurite outgrowth increases in response to optical stimulation. (a–d) Representative confocal images of DRGs stained for neurofilament (red): (a) a stimulated ChR2-DRG, (b) ChR2-DRG without stimulation, (c) Stimulated WT DRG, (d) WT DRG without stimulation. Scale bars = 2 mm. (e–h) High-resolution confocal images demonstrate ChR2 expression in (e–f) ChR2-DRGs, and no expression in (g, h) WT DRGs (Scale bars = 50 μm). (i) Confocal neurofilament image (red) is overlaid with computer-generated end points (green) for the neurite extension to illustrate the algorithm for determining the coverage area and the maximum neurite outgrowth. Neurite coverage area is determined as the area of the polygon connecting the end points generated by 360 cross-sectional profiles (blue line) separated by 1°. Maximal outgrowth is determined by the most-distant end point. (j) Pixel intensity profile (blue) obtained by one of the 360 cross-sectional profiles from the center of the DRG in (i). The end points of neurite extension (green circles) are 1σ above the average fluorescence intensity (red line). (k,l) The mean values of neurite coverage area (k) and the maximum neurite extension (l) for stimulated and unstimulated ChR2-DRGs, and stimulated and unstimulated WT DRGs. Error bars represent standard deviation (n = 6, * *p* < 0.05, ** *p* < 0.01, one-way ANOVA and Tukey's comparison test).

The overall area covered by extending neurites as well as the maximum extent of growth was significantly increased for optically stimulated *Thy1-ChR2-YFP* DRGs (ChR2-DRGs) as compared to stimulated WT DRGs, unstimulated ChR2-DRGs, and unstimulated WT DRGs (Fig. 2). For stimulated ChR2-DRGs, neurite coverage area was found to be 40.4 ± 8.9 mm^2^ (mean ± SD), a 3.3, 2.4, and 3.2 fold increase as compared to unstimulated ChR2-DRGs (12.4 ± 4.1 mm^2^), stimulated WT (17.1 ± 7.0 mm^2^), and unstimulated WT controls (12.4 ± 7.0 mm^2^) respectively (Fig. 2i). The longest neurite extension of 3.9 ± 0.6 mm was similarly observed for stimulated ChR2-DRGs, corresponding to a 1.7, 1.5, and 1.5-fold increase over unstimulated ChR2-DRG, stimulated WT DRG, and unstimulated WT DRG controls (Fig. 2h). Our observations were also consistent with AC electrical stimulation performed according to the identical paradigm with the exception of pulse width, which was chosen to be 100 μs in agreement with previous reports^33^. AC electrical stimulation at 20 Hz has yielded neurite outgrowth in ChR2-DRGs and WT DRGs (3.2 ± 0.5 mm and 3.0 ± 0.30 mm) greater than in unstimulated controls and lower than in optically stimulated ChR2-DRGs.

### Identification of optical stimulation parameters for maximum neurite outgrowth

**Figure 3:**
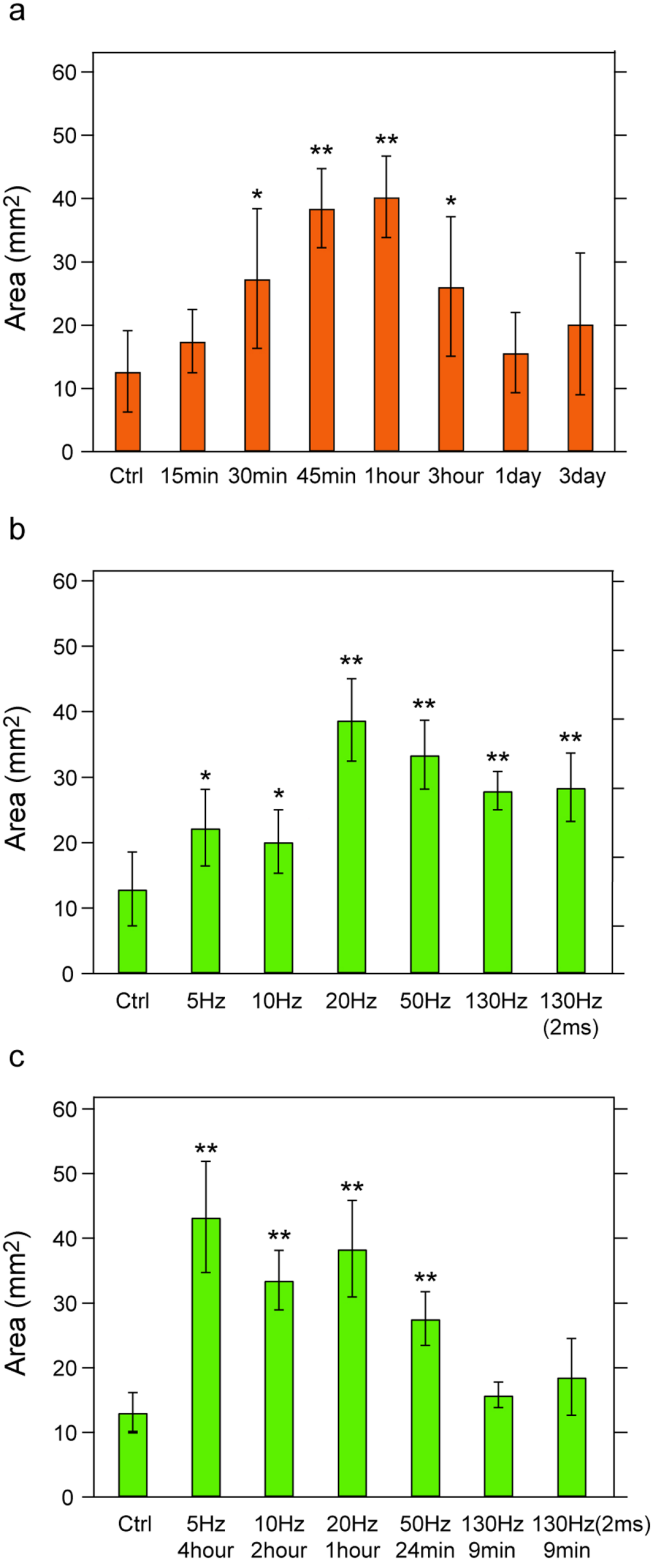
Number of light pulses is critical for neurite outgrowth. (a) The mean area of neurite coverage as a function of stimulation duration for a constant frequency (20 Hz). (b) The mean area of neurite coverage with increasing stimulation frequency for a fixed duration (1 hour). (c) Neurite coverage area for increasing frequencies at varied optical stimulation durations to deliver a constant number of light pulses (36,000). Error bars represent standard deviation (n = 6, * *p* < 0.05, ** *p* < 0.01, one-way ANOVA and Tukey's comparison test).

To identify effective stimulation conditions for promoting growth, we examined the influence of duration and frequency of optical excitation. Keeping the frequency and pulse width constant at 20 Hz and 5 ms respectively, we varied the duration of stimulation between 15 min and 3 days. Neurite outgrowth, as quantified by the total coverage area, increased for stimulation epochs between 15 and 45 min, reaching a plateau between 45 min and 1 hour. Longer stimulation periods, 3 hrs–3 days, did not yield enhanced growth, as compared to unstimulated ChR2-DRG controls (Fig. 3a).

Alternatively, maintaining the duration of stimulation at 1 hour and the pulse width at 5 ms, we examined the effects of stimulation frequency (5–130 Hz) on the neurite coverage (Fig. 3b). A positive influence on growth was observed for all stimulation frequencies, which was most pronounced between 20 Hz and 130 Hz. However, consistent with findings associated with electrical stimulation, the maximal outgrowth was observed for 20 Hz (38.8 ± 6.3 mm^2^, a 3.0-fold increase as compared to unstimulated and WT controls, *p* = 0.0006). As stimulation at 130 Hz with 5 ms pulses results in a comparatively high duty cycle, stimulation with 2 ms pulses was additionally explored at this frequency. No statistical difference in neurite outgrowth was found between the two stimulation conditions (Fig. 3b, *p* = 0.84).

We hypothesized that the apparent differences in neurite outgrowth corresponding to varied frequency and duration of optical stimulation stem from a difference in the total number of the delivered optical pulses. To test this, both the frequency and duration of stimulation were adjusted to deliver a fixed number of light pulses (36,000) that corresponded to maximum outgrowth conditions, 1 hour at 20 Hz (1 second bursts, 1 second rest epochs). Specifically, this constraint was reflected in a change in stimulation duration for frequencies of 5 Hz (4 hours), 10 Hz (2 hours), 50 Hz (24 min), and 130 Hz (9 min) used for the experiment. For a fixed number of stimulation pulses, maximum outgrowth was observed at 5 Hz (43.3 ± 8.6 mm^2^, 3.3-fold, p < 0.001), which was not significantly different from 10 Hz (33.5 ± 4.6 mm^2^) or 20 Hz. (38.4 ± 7.5 mm^2^). Comparatively lower neurite outgrowth was observed for 50 Hz (27.6 ± 4.1 mm^2^), and no difference was found for 130 Hz (either 5 ms or 2 ms pulse width) as compared to unstimulated controls and WT controls (Fig. 3c).

### Population-specific stimulation of *Thy1-ChR2-YFP* DRGs in the presence of WT DRGs

**Figure 4:**
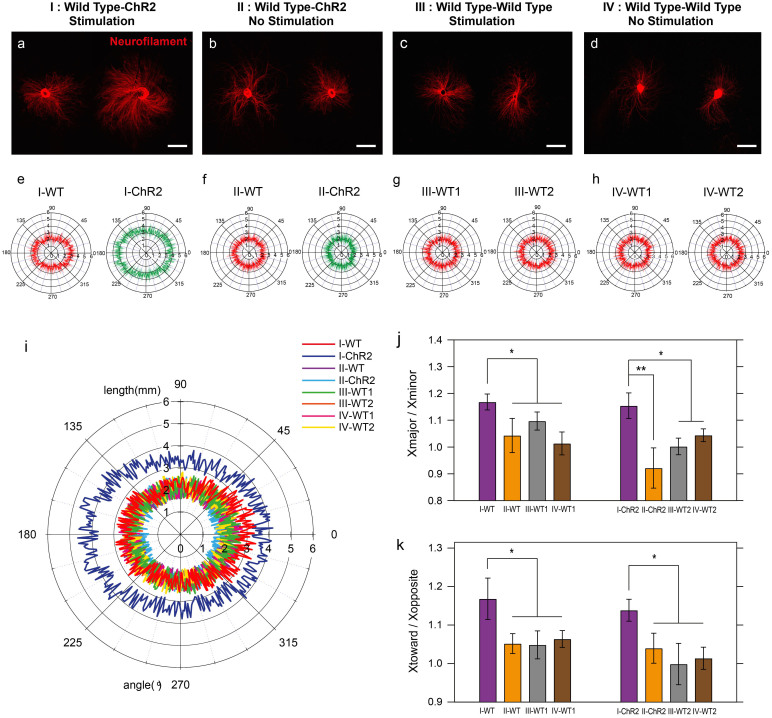
Co-cultured ChR2-DRGs and WT DRGs exhibit a directional increase of neurite outgrowth in the presence of optical stimulation. (a–d) Confocal microscopy images of neurite outgrowth (neurofilament, red). (a) Case I: co-culture of ChR2-DRG – WT DRG with stimulation (1 hour, 20 Hz, 5 ms pulse width). (b) Case II: co-culture of ChR2-DRG – WT DRG without stimulation. (c) Case III: co-culture of WT – WT DRG with stimulation. (d) Case IV: co-culture of WT – WT DRG without stimulation (scale bars = 2 mm). (e–h) Polar graphs representing the average shape of DRGs for (e) case I, (f) case II, (g) case III, and (h) case IV. All ChR2-DRGs were identified by their expression of YFP and all images were oriented with the ChR2-DRG on the right, and the DRG centers aligned on the x-axis. (i) Combined polar graph for mean outlines of both “left” and “right” DRGs for each of the four cases (8 scenarios: case I – WT, case I – ChR2, case II – WT, case II – ChR2, case III – WT1, case III – WT2, case IV – WT1, case IV – WT2). (j) Neurite outgrowth areas were fit with ellipses, yielding a major and minor axis. The length ratio between major (X_major_) and minor axes (X_minor_) of the DRG ellipses were compared to examine the directionality of system. (k) The ratio of mean outgrowth lengths between directions toward (X_toward_) and opposite (X_opposite_) to the neighboring DRG. Error bars represent standard deviation (n = 6, * *p* < 0.05, ** *p* < 0.01; one-way ANOVA and Tukey's comparison test).

Using ChR2-DRGs from *Thy1-ChR2-YFP* mice enabled population selectivity of optogenetic stimulation to investigate whether optically evoked activity would alter the growth of a neighboring unstimulated, WT DRG. Four cases of paired outgrowth were examined: Case I: WT DRG – ChR2-DRG with 20 Hz, 1 hour stimulation (Fig. 4a), Case II: WT DRG – ChR2-DRG without stimulation (Fig. 4b), Case III: WT – WT with stimulation (Fig. 4c), Case IV: WT – WT without stimulation (Fig. 4d). DRGs were consistently placed 10.1 ± 0.8 mm apart (no significant difference between sample preparations), a substantial distance commonly used in rodent models of peripheral nerve transection injury^34^,^35^. Consistent with the findings for lone DRGs, we observed increased outgrowth from ChR2-DRGs subjected to optical stimulation. Additionally, in the presence of optically stimulated ChR2-DRG, the neurite outgrowth of a neighboring WT DRG was enhanced (Fig. 4d, g) and exhibited a directional preference towards the ChR2-DRG (Fig. 4h–i). Concomitantly, neurite outgrowth from ChR2-DRGs was directionally biased towards corresponding WT DRGs (Fig. 4h–i), suggesting a complex interplay of soluble factor expression, paracrine signaling, and neurite chemotaxis^13^,^15^. For control Cases II, III, and IV, no increase in outgrowth or directional bias was observed.

### Effects of optical stimulation on expression of neurotrophic factors

**Figure 5:**
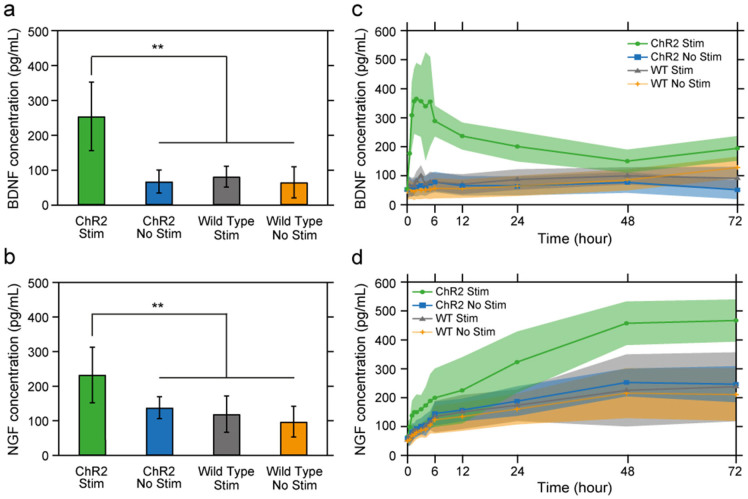
BDNF and NGF expression increased with optical stimulation. (a–b) The mean concentrations of released BDNF (a) and NGF (b) for stimulated and unstimulated ChR2-DRGs, and WT DRGs. The parameters of optical stimulation were fixed to 1 hour, 20 Hz, 5 ms pulses. Error bars represent standard deviation (n = 3, * p < 0.05, ** p < 0.01, one-way ANOVA and Tukey's comparison test). (c–d) The mean concentration of BDNF and NGF (solid lines) in the cell-culture media as a function of time (Every hour for 0–6 hours, 12, 24, 48, and 72 hours) for stimulated and unstimulated ChR2-DRGs and WT DRGs. Shaded areas represent standard deviation.

To test the hypothesis that optical stimulation increases the release of soluble neurotrophic factors, we used ELISA assays to measure the concentrations of BDNF and NGF excreted from stimulated (20 Hz, 1 hour) and unstimulated ChR2-DRG and WT DRG for 72 hours following stimulation (Fig. 5). BDNF and NGF concentrations of stimulated ChR2-DRG (260.3 ± 98.3 pg/mL and 215.0 ± 98.2 pg/mL) exhibited a 4.0, 3.4, and 4.0-fold and 1.6, 1.8, and 2.2-fold increase as compared to unstimulated ChR2-DRGs (64.4 ± 32.9 pg/mL and 130.8 ± 31.5 pg/mL) and stimulated (76.7 ± 30.0 pg/mL and 121.9 ± 52.4 pg/mL), and unstimulated WT (65.1 ± 44.8 pg/mL and 97.5 ± 45.2 pg/mL) controls, respectively (Fig. 5a, b). Closer examination of the temporal dynamics of neurotrophic factor release revealed that the concentration of BDNF increased in the first 2 hours following optogenetic stimulation, reaching a plateau around 400 pg/mL, followed by a subsequent decay 5 hours after stimulation. No significant changes in BDNF concentration were observed for unstimulated ChR2-DRGs, stimulated and unstimulated WT controls (Fig. 5c). In contrast, the concentration of NGF continuously increased over time for all DRG samples, however a significantly greater increase was observed for stimulated ChR2-DRGs (Fig. 5d).

### Schwann cell migration during optical stimulation

**Figure 6:**
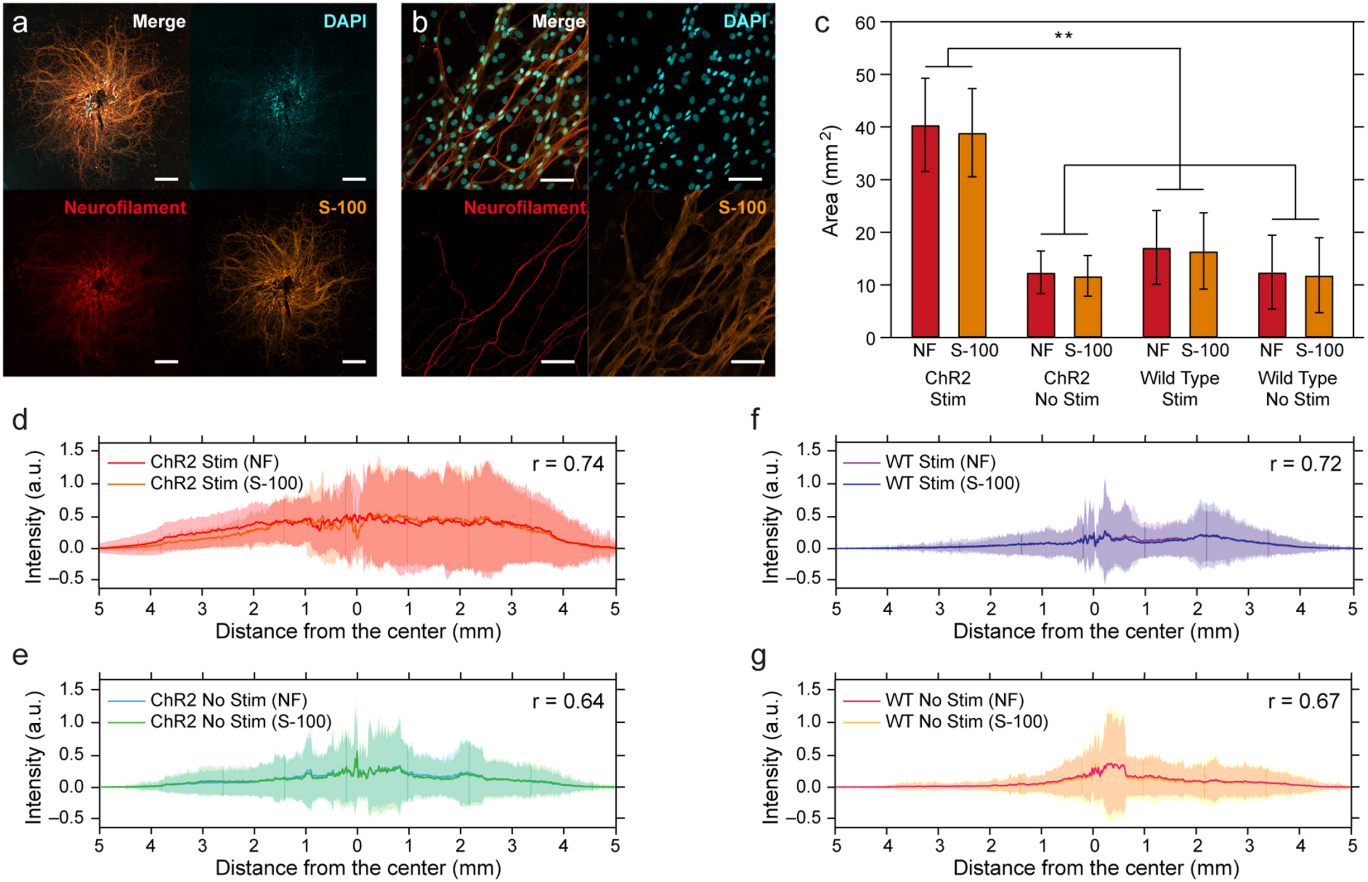
Schwann cell migration follows increased neurite outgrowth in the presence of optical stimulation. (a) Representative confocal images for whole DRGs stained for DAPI (cyan), neurofilament (red), and S-100 (a marker of Schwann cells, gold) (scale bars = 1 mm). (b) Higher resolution confocal images (40× objective) of DAPI (cyan), neurofilament (red), and S-100 (gold) (scale bars = 50 μm). (c) The mean value of coverage area of neurofilament (NF, red) and Schwann cells (S-100, orange) for stimulated and unstimulated ChR2-RGs and WT DRGs. Error bars represent standard deviation (n = 6, * *p* < 0.05, ** *p* < 0.01; one-way ANOVA and Tukey's comparison test). (d–g) Fluorescence intensity profiles for neurofilament and Schwann cells for stimulated ChR2-DRGs (d), unstimulated ChR2-DRG (e), stimulated WT DRG (f), and unstimulated WT DRG (g). Intensity (a.u.) is the relative pixel intensity, and shaded areas represent standard deviation.

According to recent studies, a rise in the NGF concentration of stimulated DRG cultures is consistent with increased migration of Schwann cells^20^. Similarly to the extent of neurite outgrowth, we found that the migration of Schwann cells was increased for optically stimulated ChR2-DRGs as compared to unstimulated ChR2-DRGs and WT controls (Fig. 6a–c). Schwann cells were collocated with neuronal processes for all four conditions (stimulated and unstimulated ChR2-DRGs and WT controls), as confirmed by the overlap of fluorescence intensity profiles as well as the high Pearson Correlation Coefficients (stimulated ChR2-DRGs: PCC = 0.74, unstimulated ChR2-DRGs: 0.64, stimulated WT DRGs: 0.72, unstimulated WT DRGs: 0.67) corresponding to Neurofilament and S-100 immunostained images (Fig. 6d–g).

## Discussion

In this report, we demonstrated the application of optogenetics to enhance neurite outgrowth from peripheral neural tissue expressing ChR2 in a population-specific fashion as an alternative to AC electrical stimulation. Optogenetic stimulation is additionally advantageous for its compatibility with electrophysiological recordings of neural regeneration in injury models.

The increased rate of axonal regeneration in response to AC electrical stimulation has been previously correlated with changes in the expression profiles of BDNF, TrkB receptor, and regeneration-associated proteins Talpha1-tubulin and GAP-43^19^,^36^,^37^. It has been demonstrated that neural activity results in rapid release of BDNF^38^ and recruitment of TrkB receptors to the cell membrane^39^. In addition, AC stimulation has no effect on nerve growth when sodium ion influx *via* voltage gated channels was blocked by an infusion of tetrodotoxin^40^. Taken together, this previous work indicates that the membrane depolarization caused by electrical stimulation impacts the BDNF signaling pathway, and thus neural regeneration. Our experiments indicate that optical stimulation of ChR2 expressing tissues produces an even greater enhancement of neurite outgrowth as compared to AC stimulation, and similar mechanisms for promoting neurite growth may be triggered by both stimulation modalities. This hypothesis is supported by an increase in NGF and BDNF concentration following optogenetic stimulation of ChR2-DRGs (Fig. 5). Previous work has indicated that AC electrical stimulation induces a calcium ion influx and externalization of both TrkB and BDNF, influencing TrkB-mediated neurotrophin signaling^41^. The non-monotonic dependence of outgrowth enhancement on the duration of stimulation remains unclear, however Geremia *et al.* have hypothesized that prolonged stimulation yields desensitization of sensory neurons *via* down-regulation of TrkB receptor^40^.

Our custom designed LED array provided a convenient platform for rapid screening of a broad range of stimulation parameters to identify the effective conditions for promoting neurite growth. We initially observed maximum enhancement of outgrowth for 1 hour of optical excitation at 20 Hz – consistent with the results of AC electrical stimulation^42^. However, further investigation revealed that the number of optical pulses, rather than frequency or duration, is the predominant cue for promoting neurite growth. In fact, at a fixed number of stimulation pulses, the largest extent of outgrowth was exhibited at lower frequencies. This trend (Fig. 3c) is consistent with the recent study by Mattis *et al.*, who showed that the likelihood of firing an action potential by ChR2 expressing neurons in response to optical pulses decreased at higher frequencies^43^. This response is typically attributed to the emergence of a plateau potential at high stimulation frequencies that impairs membrane repolarization by voltage-dependent ion channels^44^.

In addition to maximizing total growth, effective reinnervation of distal targets in the PNS following injury requires directional control of regenerating axons' growth cones. Our results suggest that population-specific optogenetic stimulation may provide a strategy to directionally control outgrowth by creating concentration gradients of neurotrophic factors (Fig. 4). The directional bias of neurite outgrowth in our co-cultures suggests chemotaxis of neurites in response to gradients of soluble factors produced by optical stimulation of ChR2-DRGs. Since the DRGs were placed 10 mm apart, formation of synaptic connections between them is unlikely and was not observed in our experiments. As the distance of 10 mm is comparable to a large-gap PNS injury in a rodent model^34^,^35^, the proposed approach may provide further insight into the mechanisms involved in directing neural regeneration with population specificity inaccessible with AC electrical stimulation.

We observed that the migration of Schwann cells accompanies neurite extension (Fig. 6). Consequently, optically stimulated ChR2-DRGs exhibit the largest outgrowth, and their associated Schwann cell networks were similarly expanded. It is unclear, however, whether the neuronal depolarization evoked by optical stimulation yields the expansion of the Schwann cell networks or if Schwann cell migration is modulated by light directly. The increasing concentration of NGF in stimulated ChR2-DRG cultures, however, is consistent with Schwann cell supportive behavior (Fig. 5c–d, Fig. 6). Future experiments involving viral delivery of opsins under promoters specific to glia may further elucidate the role of Schwann cells in the context of optical stimulation^45^,^46^.

In conclusion, our data demonstrates that pulsed optogenetic stimulation promotes neurite outgrowth from *Thy1-ChR2-YFP* DRGs *in vitro*. Our custom light-delivery platform provided a high-throughput screening tool to identify effective simulation parameters. Specifically, we found that the number of optical pulses is the major parameter accelerating neurite growth. Population-specific application of optogenetic stimulation in a DRG co-culture enabled us to demonstrate activity-dependent, directional bias of neurite outgrowth. Our findings suggest that optical stimulation provides a cell-specific alternative to electrical stimulation, allowing for exploration of underlying electrophysiological mechanisms of axonal growth and aiding in the design of future regenerative therapies for PNS injuries.

## Methods

### LED stimulation array

To deliver pulsed blue light to optically sensitive tissue, 12 Blue light-emitting diodes (LEDs, emission peak λ = 465 nm, Cree, Durham, NC) were soldered to a custom designed printed circuit board (PCB) (ExpressPCB, Santa Barbara, CA). LEDs were driven by an Arduino Uno (Newark, Palatine, IL) connected to the PCB at prescribed durations and frequencies as coded in the Arduino environment software. A compact power and energy meter (PM100D, S130C, Thor Labs, Newton, NJ) was used to measure light intensity. Growth media temperature was monitored in an experimental setup without DRGs for 12 hours via a non-conductive fluorescence thermometer (HHTFO-101, Omega Engineering, Stamford, CT) for light pulses at 5 Hz, 20 Hz, 50 Hz, and 130 Hz.

### Dorsal root ganglion (DRG) isolation

All animal procedures were approved by the MIT Committee on Animal Care and carried out in accordance with the National Institutes of Health Guide for the Care and Use of Laboratory Animals. 35 *Thy1-ChR2-YFP* and 15 WT mice were used in this study. A litter of neonates typically consisted of 3–5 animals. DRGs were extracted from P0 neonatal transgenic *Thy1-ChR2-YFP* and wild-type (WT) mice. The spinal cord was exposed using a posterior approach and the vertebral bodies were removed. Individual DRG explants were trimmed of nerve roots and connective tissue and placed within one stock container of growth media. On average, 10–15 DRGs were extracted from each animal and mixed. From the solution containing 30–70 DRGs from 3–5 animals we randomly picked n = 6 DRGs per experimental condition, plated on matrigel-coated coverslips, and incubated (37°C, 5% CO_2_). Round glass coverslips (12 mm Electron Microscopy Sciences, Hatfield, PA) were acid etched with 10% HCl solution (Sigma, St. Louis, MO) and then washed and stored in 99% ethanol for use on demand. Coverslips were dried and placed into 12-well tissue culture plates (VWR Scientific Products, Edison, NJ) and coated with a 1:30 dilution of reduced growth factor Matrigel® (BD Bioscience, San Jose, CA) in Neurobasal A-medium (Life Technologies, Grand Island, NY) supplemented with B-27 (Life Technologies), 5 ml Glutamax-I (Life Technologies) and 1 ml of pen/strep (Lonza, Basel, Switzerland). All DRGs were grown for 48 hours prior to applying optical stimulation and fixed 12 days following seeding.

### Optical stimulation protocols

Extracted *Thy1-ChR2-YFP* and WT DRGs (n = 6 per test condition as described above) were stimulated with blue light at 20 Hz for 1 hour. Optical stimulation was applied 48 hours following seeding to allow for robust DRG attachment. An unstimulated control group (ChR2-DRG, n = 6) were cultured simultaneously, but not subjected to optical stimulation. To find the effective characteristics of optical stimulation, a broad range of pulse durations and frequencies was evaluated. ChR2-DRGs (n = 6) were stimulated at a constant frequency for 15 min, 30 min, 45 min, 1 hour, 3 hours, 1 day, and 3 days, respectively. Alternatively, stimulation duration was held to 1 hour and frequencies of 5, 10, 20, 50, or 130 Hz were delivered. All light pulses were kept constant at 5 ms and all stimulation paradigms consisted of 1 s of stimulation followed by 1 s of rest, repeated for the full duration of the stimulation. Only for the 130 Hz case, an additional pulse width of 2 ms was investigated. Finally, using the 1 hour, 20 Hz parameters as a standard, pulse frequency and stimulation duration were systematically altered to deliver a constant number of total pulses (36,000 pulses). This constraint was applied at the following settings: 5 Hz for 4 hours, 10 Hz for 2 hours, 20 Hz for 1 hour, 50 Hz for 24 min, 130 Hz for 9 min, and 130 Hz (2 ms pulse length) for 9 min (n = 6 per setting).

### Electrical stimulation

AC Electrical stimulation was applied to Thy1-ChR2-YFP and WT DRGs (n = 6 per test condition) after 48 hours of seeding which allowed for robust DRG attachment. For stimulation, two platinum electrodes (Sigma) were inserted into the culture medium for providing an electric field across the length of the entire well. Electrical pulses (3 V, 20 Hz, 100 μs pulse width) were applied by the Arduino circuit with voltage dividers. The stimulation pattern consisted of alternating 1 s epochs of stimulation and rest, and was repeated during 1 hour.

### Population-specific stimulation in DRG co-cultures

To investigate the influence of an optically stimulated ChR2-expressing DRG on the outgrowth of a WT DRG in a co-culture system, a stimulation protocol similar to that described above was followed. However, two DRGs were seeded on the same coverslip separated by 10 mm. Three different combinations of DRG genotypes and optical stimulation were investigated (n = 6 per case; Fig. 4): Case I, WT DRG and ChR2-DRG with stimulation (20 Hz for 1 hour); Case II, WT DRG and ChR2-DRG with no stimulation; Case III, two WT DRGs with stimulation (20 Hz for 1 hour); and Case IV, WT DRGs with no stimulation.

### Immunocytochemistry

DRGs were fixed 10 days following the onset of optical stimulation with 4% (Wt. %) paraformaldehyde (Sigma) solution in phosphate buffered saline (PBS, VWR) for 25 min and permeabilized with 0.1% Triton X-100 (Sigma) in PBS for 10 min at room temperature. After blocking overnight with 2.5% goat serum (Sigma) solution in PBS at 4°C, samples were incubated with primary antibodies, 1:500 rabbit anti neurofilament antibody (N4142, Sigma) and 1:500 rat anti S-100 antibody (S2532, Sigma) solution in 2.5% goat serum solution in PBS for 1 hour. Samples were then incubated with secondary antibodies, 1:1000 Alexa Fluor® 633 goat anti-rabbit IgG (A-21070, Life Technologies) and 1:1000 Alexa Fluor® 568 goat-anti-rat IgG (A-11077, Life Technologies) in 2.5% goat serum in PBS for 1 hour. Lastly, samples were incubated with 1:10,000 DAPI (D9542, Sigma) solution in PBS for 15 min and mounted on microscope slides in ProLong® Gold Antifade reagent (P36941, Life Technologies).

### Microscopy and image analysis

Images were acquired with an Olympus FV1000 laser scanning confocal microscope (Olympus). Overview mosaic images were taken with an air-immersion 4× objective (Olympus) and the higher magnification images were taken with a water-immersion 40× objective (Olympus). A custom image analysis algorithm written in MATLAB (Mathworks, Natick, MA) was used to quantify the total neurite outgrowth coverage area as well as the maximum outgrowth length. Specifically, 360 equally distributed radial lines, centered in the middle of the DRG were overlaid onto the image. Pixel intensity values, which are the values relative to the maximum pixel intensity in the figure, were found along each line, and the extent of neurite outgrowth was determined as 1σ standard deviation above the average (Fig. 2j). Two corresponding end points for neurite extension were found for each radial line and the total coverage area was calculated as the area within a 2-D outline of all end points (720, 2 per line). The longest extent of growth was defined as the distance from the center of the DRG to the most distant end point.

### ELISA assay

The concentrations of nerve growth factor (NGF) and brain-derived neurotrophic factor (BDNF) were measured for stimulated and unstimulated ChR2-DRGs as well as stimulated WT DRGs using a mouse NGF and BDNF ELISA Kit (Insight Genomics, Falls Church, VA). Mouse NGF/BDNF polyclonal antibodies were pre-coated onto a 96 well plate. After washing and 2 hours of incubation, different concentrations of NGF and BDNF (31.2, 62.5, 125, 250, 500, 1000, 2000 pg/ml), samples from the DRG cultured media (for 0, 1, 2, 3, 4, 5, 6, 12, 24, 48, 72 hours), and biotinylated detection antibodies were added to the well and washed with the provided buffer. Avidin-Biotin-Peroxidase Complex was added to bind the biotinylated detection antibodies, and then 3, 3′, 5, 5′-Tetramethylbenzidine (TMB), and a horseradish peroxidase (HRP) substrate was used to visualize HRP enzymatic reaction. Optical absorbance was measured using a Varioskan Flash Reader (Thermo Fisher Scientific, Waltham, MA) at 450 nm, and the concentration was calculated using provided calibration standards.

### Statistical analysis

Group size was determined by a power analysis in Matlab using the sampsizepwr function as implemented in the statistics toolbox, estimating that optogenetic stimulation should double the outgrowth length as compared to WT controls without stimulation (α = 0.05, power = 0.9). Statistical significance was assessed by first ensuring normal distribution and comparable variance of the results via Lilliefors and Bartlett's test, respectively, followed by a one-way ANOVA and Tukey's post-hoc comparison test. For direct comparison between two cohorts we used one-sided Student's t-tests.

## Author Contributions

S.P., R.A.K., B.L.M. and P.A. designed the study. S.P. and R.A.K. performed the experiments. S.P., R.A.K., U.P.F., X.J., A.K.H.A. and P.A. analyzed the data. All authors have contributed to writing the manuscript.

## Additional Information

**How to cite this article**: Park, S. *et al.* Optogenetic control of nerve growth. *Sci. Rep.* 5, 9669; DOI:10.1038/srep09669 (2015).
